# Pain sensitivity differs between dog breeds but not in the way veterinarians believe

**DOI:** 10.3389/fpain.2023.1165340

**Published:** 2023-06-26

**Authors:** Rachel M. P. Caddiell, Rachael M. Cunningham, Philip A. White, B. Duncan X. Lascelles, Margaret E. Gruen

**Affiliations:** ^1^ Comparative Behavioral Research, Department of Clinical Sciences, College of Veterinary Medicine, North Carolina State University, Raleigh, NC, United States; ^2^ Translational Research in Pain, Department of Clinical Sciences, College of Veterinary Medicine, North Carolina State University, Raleigh, NC, United States; ^3^ Department of Statistics, College of Physical and Mathematical Sciences, Brigham Young University, Provo, UT, United States; ^4^ Comparative Pain Research and Education Center, College of Veterinary Medicine, North Carolina State University, Raleigh, NC, United States; ^5^ Thurston Arthritis Centre, UNC School of Medicine, Chapel Hill, NC, United States; ^6^ Center for Translational Pain Research, Department of Anesthesiology, Duke University, Durham, NC, United States

**Keywords:** healthcare provider beliefs, attitudes, stereotypes, perceptions of patient pain, canine behavior, quantitative sensory testing, animal welfare, affective states

## Abstract

**Background:**

Veterinarians hold distinct breed-specific pain sensitivity beliefs that differ from the general public but are highly consistent with one another. This is remarkable as there is no current scientific evidence for biological differences in pain sensitivity across dog breeds. Therefore, the present study evaluated whether pain sensitivity thresholds differ across a set of dog breeds and, if so, whether veterinarians’ pain sensitivity ratings explain these differences or whether these ratings are attributed to behavioral characteristics.

**Methods:**

Pain sensitivity thresholds [using quantitative sensory testing (QST) methods] and canine behaviors (using owner questionnaires and emotional reactivity tests) were prospectively measured across selected dog breeds. Adult, healthy dogs from 10 dog breeds/breed types were recruited, representing breeds subjectively rated by veterinarians as high (chihuahua, German shepherd, Maltese, Siberian husky), average (border collie, Boston terrier, Jack Russell terrier), or low (golden retriever, pitbull, Labrador retriever) pain sensitivity. A final sample of 149 dogs was included in statistical analyses.

**Results:**

Veterinarians’ pain sensitivity ratings provided a minimal explanation for pain sensitivity thresholds measured using QST in dogs; however, dog breeds did differ in their pain sensitivity thresholds across the QST methods evaluated. Breed differences were observed for some aspects of emotional reactivity tests; however, these behavioral differences did not explain the differences in pain sensitivity thresholds found. Veterinarians’ pain sensitivity ratings were positively associated with dog approach scores for the disgruntled stranger test suggesting that the way dogs greet strangers may be a factor influencing veterinarians’ ratings of pain sensitivity across dog breeds.

**Conclusions and clinical relevance:**

Overall, these findings highlight a need to investigate biological mechanisms that may explain breed differences in pain sensitivity because this may inform pain management recommendations. Further, future research should focus on when and how these breed-specific pain sensitivity beliefs developed in veterinarians, as veterinarians’ beliefs could impact the recognition and treatment of pain for canine patients.

## 1. Introduction

Canine pain sensitivity is widely believed to differ among dog breeds. A study conducted by Gruen et al. (1) surveyed more than 1,000 U.S. veterinarians and 1,000 members of the general public and found that most participants reported agreement with the statement, “Dog breeds differ in their sensitivity to pain.” In fact, more than 98% of veterinarians in the study's sample supported this belief. Interestingly, when asked to rate pain sensitivity for 28 different dog breeds, there were marked differences between breed-specific pain sensitivity ratings reported by members of the general public compared to veterinarians. General public breed-specific pain sensitivity ratings were primarily related to dog size (with smaller and lighter dogs being rated as having higher pain sensitivity) and the presence of breed-specific legislation (i.e., laws that regulate and/or ban certain dog breeds), whereas veterinarians reported breed-specific pain sensitivity ratings that were different from the general public but highly consistent with one another. This finding is remarkable because there is no existing scientific evidence for biological differences in pain sensitivity among dog breeds.

Veterinarians’ beliefs about dog breed pain sensitivity hold importance because they could impact pain recognition and treatment. In human medicine, researchers have identified how healthcare providers’ perceptions of patients impact their assessment of patient pain (2–4) and, therefore, their treatment decisions (5–7). Healthcare providers’ perceptions regarding patient pain sensitivity are influenced by observable phenotypic characteristics including perceived race, ethnicity, and gender (3, 4, 8). Therefore, it is imperative to understand whether veterinarians’ breed-specific pain sensitivity beliefs align with measures of pain sensitivity in dogs. If dog breeds do differ in their experience of pain, this information could guide future development of pain-scoring systems and treatment recommendations for dogs, as breeds have not previously been considered in analyses of studies evaluating and validating pain scales or assessing analgesic efficacy. Thus, understanding whether breed-related differences in pain sensitivity exist has the potential to optimize pain management in dogs. However, if breeds do not differ in pain sensitivity or their pain sensitivity differences do not align with veterinarians' beliefs, then it would be critical to understand when and how veterinarians developed these distinct breed-specific beliefs about pain sensitivity.

In both humans and animals, quantitative sensory testing (QST) methodologies are noninvasive, semi-objective research tools commonly used to evaluate the response of the somatosensory system to a standardized stimulus in a laboratory setting. When applied to a neutral or pain-free location, QST evokes a somatosensory response through the nociceptive pain pathway. Using QST, a noxious stimulus (e.g., mechanical—puncture or deep pressure, thermal—heat or cold) is applied to the skin, which is detected by nociceptors or pain receptors, a group of specialized sensory neurons that communicate information by sending nerve signals to the spinal cord and brain where pain perception occurs (9). In humans, a QST trial is considered complete when the person verbally indicates that they have reached their threshold or have experienced discomfort (10). However, in animals, the stimulus is immediately removed when the animal, in this case, a dog, indicates a behavioral response (e.g., pulls their paw away, vocalizes, and/or turns to look at the stimulus). Multiple QST modalities and methods exist to assess different noxious stimuli, as each stimuli-responsive pathway is different (e.g., different nociceptors, different afferent nerves) (11).

Quantitative sensory testing is an ideal method to assess pain sensitivity across dog breeds. Prior studies have demonstrated that QST can detect differences in pain sensitivity thresholds in dogs diagnosed with osteoarthritis pain and healthy, pain-free dogs (12, 13). Additionally, a single study conducted in New Zealand detected breed differences in pain sensitivity thresholds using thermal QST in laboratory-housed dogs (14). These findings are intriguing; however, there is a need to evaluate breed differences in a broader context by including dog breeds rated by veterinarians as either more or less sensitive to pain, dog breeds that distinctly differ in size, and pet dogs rather than laboratory-housed dogs and by balancing sex within dog breeds. Additionally, it is necessary to comprehensively assess pain sensitivity thresholds across dog breeds using multiple QST methods.

In addition to evaluating pain sensitivity thresholds, it is critical to determine whether there are any differences in behavior among dog breeds and, if so, whether these differences influence measures of pain sensitivity. This is particularly important because veterinarians reported that dog temperament has the greatest influence on their pain sensitivity ratings; this was above genetics, developmental environment, and skin thickness (1). Therefore, it is possible that veterinarians may be identifying behavioral reactivity differences among dog breeds rather than differences in the actual sensation of pain.

A combined approach to measuring canine behavior by using both clinical metrology instruments (CMIs) (validated questionnaires) and emotional reactivity tests (ERTs) is ideal to achieve a thorough understanding of breed differences. Since dogs cannot self-report their subjective states (e.g., pain, fear, anxiety), observations of their behaviors by individuals who are familiar to them can be relied on as a proxy assessment. CMIs are used by researchers to ascertain information from owners about their dogs’ affective states that may confound pain, such as anxiety or fear. Additionally, owners can be a valuable source of information about their dog's behavior because they observe the dog daily and in varied contexts; thus, they can provide a comprehensive view of their dog's behavior over time. The Canine Behavioral Assessment and Research Questionnaire (C-BARQ) is a validated, reliable 100-item CMI (15–18) that has previously been used to identify behavioral differences (including differences in aggression, fear and anxiety, trainability, and touch sensitivity) among dog breeds (19–21). However, solely relying on owner-reported behavior can pose challenges, as this is a secondary source of information about the dog's subjective state and depends on the owner to be vigilant in their behavioral observations, and responses are likely shaped by the owners’ perceptions and experiences. Therefore, standardized behavioral tests, termed ERTs, can be used in the laboratory setting to allow researchers to assess behavior through standardized direct observations. Emotional reactivity tests can be used to assess components of fear and anxiety that dogs may experience and distinguish these from nonpainful aspects of being in a veterinary environment, such as encountering novel stimuli and interacting with strangers. Emotional reactivity tests are standardized tasks characterized by their relatively short duration (e.g., less than 2 min) (22, 23). An advantage of using ERTs is that behaviors can be directly assessed and reviewed by trained researchers through video recordings. Behavioral analysis involves measuring the frequency, duration, or latency of behaviors that occurred (24), as well as the assignment of categorical or ordinal ratings for behavioral responses (25, 26).

The primary aim of this study was to determine whether veterinarians’ breed-specific pain sensitivity ratings explain pain sensitivity thresholds measured across dog breeds using QST. In answering this research question, we will additionally evaluate whether pain sensitivity thresholds differ across dog breeds and, if so, whether these differences are explained by dog behavior as measured through CMIs and ERTs. The null hypothesis we proposed was that dog breeds will not differ in pain sensitivity thresholds and that veterinarians’ pain sensitivity ratings are attributed to other factors, such as behavior.

## 2. Materials and methods

This research protocol was approved by the Institutional Care and Use Committee of North Carolina State University (NCSU) (20-327-O).

### 2.1. Animals

**Figure 1:**
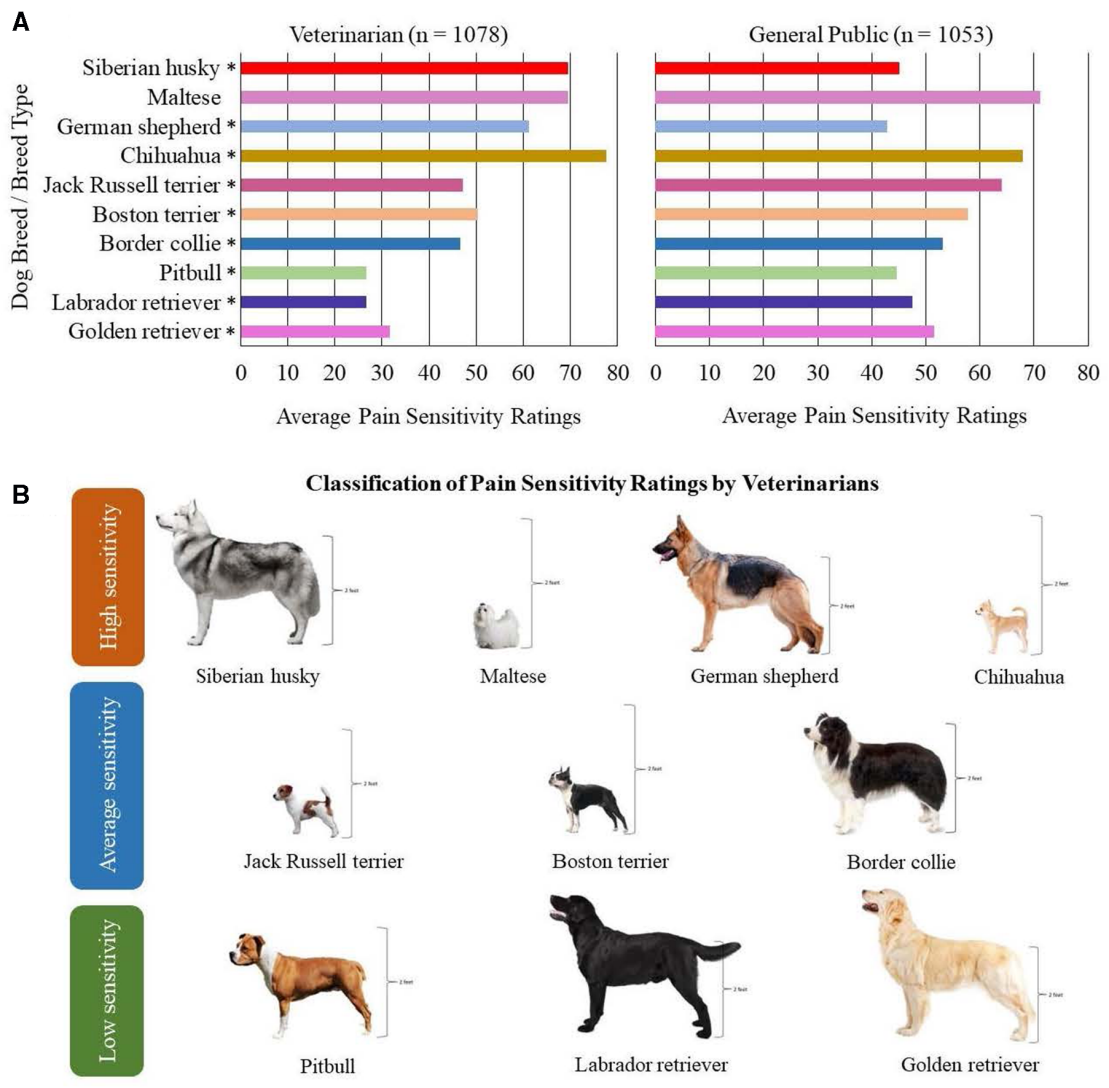
Ten dog breeds/breed types selected for study inclusion. (**A**) Findings from Gruen et al. (1) demonstrating the average pain sensitivity ratings by both veterinarians and general public members for the ten dog breeds selected. The scale ranged from 0 = not at all sensitive to 100 = most sensitive imaginable. In Gruen et al. (1), median pain sensitivity ratings between veterinarians and the general public were compared using two-sample *t*-tests, and *p*-values = 0.001 are indicated using asterisks (*). (**B**) Visual representation of the ten dog breeds/breed types selected based on the classification of pain sensitivity ratings by veterinarians. Height is demonstrated for each breed, as consideration was provided to include dog breeds/breed types of varying sizes.

Ten dog breeds/breed types were considered for inclusion in this study to encompass purebred dogs whose pain sensitivity ratings were significantly different between the veterinarian and general public populations, belonged to different classifications of pain ratings by veterinarians (e.g., rated as having high sensitivity, average sensitivity, and low sensitivity), and were of varying sizes (Figure 1) (1). Additional inclusion criteria for the study were that dogs were healthy, nonpainful (as determined through physical and orthopedic examinations and review of medical records), and of adult age status for their breed. A maximum of two dogs per household and/or genetic relation could be enrolled in the study with an exception made for a household with four Jack Russell terriers due to the limited availability of willing participants with dogs belonging to this breed.

Client-owned dogs were recruited over 11 months. Purposive sampling was conducted to recruit dogs from North Carolina and surrounding states, as owners had to be willing to transport their dog(s) to the NCSU College of Veterinary Medicine (CVM) Health and Wellness Center located in Raleigh, NC, United States, for a single day-long visit. An advertisement flyer explaining the study purpose and inclusion criteria, along with a screening survey link (developed using Qualtrics), was created to recruit interested owners. The screening survey collected information about the dog's characteristics including their breed, age, sex, and health status, as well as asked their owner permission to obtain copies of their veterinary records. Recruitment was conducted with the assistance of the Clinical Studies Core at the NCSU Comparative Medicine Institute (https://cvm.ncsu.edu/research/research-labs/clinical-studies/) using social media, email, and paper advertisements within the NCSU community and local veterinary practices in Raleigh and surrounding areas. Additional recruitment methods were required and consisted of circulating the study flyer with local breed groups, veterinarians, breeders, dog training facilities, and dog daycares, as well as on community Facebook pages. Furthermore, multiple Facebook advertisements were purchased. Snowball sampling techniques were employed because owners were encouraged to share the study flyer with other interested owners and community members. Owners were made aware that their pets would receive examinations, blood work, and a dog toy at no charge.

A sample size calculation was conducted using previously collected mechanical QST data. Using these data, we proposed that group differences between the three classifications of pain sensitivity ratings by veterinarians (high, average, and low sensitivity groups) would differ by 100 g using the blunt probed pressure algometer (PA) (described in further detail below), with a SD of 250 g. Group differences of 100 g would be considered clinically meaningful, as the presence of osteoarthritis resulted in group differences of ∼200 g. Additionally, we used data from an unaffiliated research group to estimate how many dogs per sensitivity group would be needed to detect differences. Bowden et al. (14) evaluated pain sensitivity thresholds across three breeds of dogs and were able to detect that one breed reacted at a temperature of 1.3 degrees celcius higher with a pooled SD of 1.45°. However, their methodology is not directly applicable to the present study because they used threshold temperature to elicit reactions to thermal QST, whereas our research group uses latency to react at a set temperature (further elaborated below). Combing these approaches, our standard size estimation revealed that at least 130 dogs across the three different classifications of pain sensitivity ratings, as well as 14 dogs per breed, were needed to detect group differences with a power of 0.90 and an *α* of 0.05. Dogs were selected for the study with consideration given to creating an equal sex distribution for each breed.

### 2.2. General experimental procedures

Prior to participating in the study, owners provided both verbal and written informed consent for their dogs. Owners were asked to complete a CMI to ascertain information about their dog's behavioral repertoire in their home environment. Upon arrival at the NCSU CVM Health and Wellness Center, each dog was taken to a testing room and allowed time to acclimate to both the room and the researchers. During this acclimation period, the dog was provided with treats, pets, and play time at their choosing. Once the dog was deemed comfortable, the study veterinarian (Dr. Rachel M. Cunningham, henceforth referred to as RMC) performed a physical and orthopedic examination to determine whether the dog met the inclusion criteria of being a healthy, nonpainful dog. If inclusion criteria were met, a patch of hair (2.54 cm × 2.54 cm) on the dog's metatarsus and carpus (left or right in randomized order) was shaved using clippers to facilitate sensory testing. Each dog participated in three sensory tests (described in detail below). There was a 5-min break between each QST method. Following the completion of all three sensory methods, 5 ml of blood was collected from either the cephalic or jugular vein (depending on the site the dog was most comfortable with) and dogs were provided with a break to relax and decompress and then taken outside to relieve themselves (free catch urine and feces were collected at this time, if available). After the break, dogs were brought to a new testing room for emotional reactivity testing (novel object task and disgruntled stranger test described in detail below). Dogs were once again provided with time to acclimate to the new testing room. The novel object task was performed first, followed by the disgruntled stranger test. Dogs received a 3–10-min break between ERTs. Given the potential for anxiety and emotional reactivity to affect a dog's response to sensory testing, emotional reactivity testing was always conducted at the end of the data collection day.

### 2.3. Clinical metrology instruments

Each dog owner electronically completed the C-BARQ for their dog. The C-BARQ is a validated and reliable 100-item CMI used to measure the prevalence and severity of canine behavioral problems (15–18). Specific factors from the C-BARQ that assess components of fear including stranger-directed fear, nonsocial fear, and dog-directed fear, as well as touch sensitivity (i.e., fearful responses to potentially painful procedures including veterinary examinations), were extracted and used as variables for this study. Additionally, the C-BARQ calculates a trainability score (e.g., their capacity for learning, obedience with simple commands, willingness to attend to their owner, retrieve objects, response to correction, and ability to ignore distractions) for each dog based on the owner's answers. The trainability score was extracted and used as a variable for analysis.

### 2.4. Sensory testing

All QST methods were performed following protocols as set forth by Cunningham et al. (27) to collect accurate and repeatable data. Quantitative sensory testing occurred in a 2.87 m × 3.25 m dedicated testing room. A white noise machine (DOHM DS; Marpac, Kings Grant, NC, United States) was used to minimize auditory distractions. A large yoga mat (1.83 m long, 1.22 m wide, 0.635 cm thick; Gorilla Mats Premium Large Exercise Mat; Yom Gorilla Mats, Carpinteria, CA, United States) covered the floor area where testing occurred to ensure dogs were comfortable when placed in lateral recumbency for testing. Further, a water bowl was provided, and water was always made available to the dog.

The same researcher (RMC) applied all QST methods across modalities and application sites. A handler was used to assist with positioning the dog into lateral recumbency and lightly to moderately restraining the dog, when necessary. Only female researchers were used during QST to address any experimenter and/or handler sex effects in the dogs’ QST responses. To date, experimenter and/or handler sex effects have not been demonstrated in the pain responses of dogs; however, Sorge et al. (28) found that male experimenters provoked a robust physiological stress response that resulted in stress-induced analgesia in rodents, which can affect baseline behavioral responses during testing.

To measure pain sensitivity thresholds on the right metatarsus, dogs were placed in left lateral recumbency, and vice versa. The QST devices were applied in a set order: electronic von Frey (EVF), PA, followed by the thermal probe. All QST methods were tested on the dog's metatarsus. The thermal probe was additionally tested on the dog's carpus to replicate the testing site location used in Bowden et al. (14).

Both mechanical devices (EVF and PA) used a ramping protocol that steadily applied increasing force (∼20 g/s) to the dog's metatarsus. When the dog responded to the stimulus or the maximum safety cut-off value was reached (EVF, 1,000 g; PA, 2,500 g), the stimulus was removed. Behavioral responses included the dog withdrawing their limb or moving the limb away from the stimulus, in conjunction with an indication of conscious perception (e.g., turning to look at the stimulus, ceasing panting, licking lips). Pain sensitivity thresholds were measured in grams.

The thermal probe was applied at a temperature of 49°C to the dog's metatarsus and carpus, respectively. When the dog responded to the stimulus or reached 20 s of thermode application, the stimulus was removed. The latency to respond to the thermal probe was measured in seconds. If the safety cut-off of 20 s was reached, then 20 s was recorded as the dog's pain sensitivity threshold.

For each QST method and/or application site, five trials were performed. Between each trial, the dog received a 60-s inter-trial interval. Based on previous work, the analysis of the replicate effect, and best practices outlined for interpreting QST in dogs, the highest and lowest values were removed, and the average of the three remaining values was used to calculate the dog's pain sensitivity threshold (27, 29). When interpreting mechanical and thermal QST data, lower force values and a shorter latency to respond indicate a greater sensitivity to pain, whereas higher force values and a longer latency to respond indicate a lower sensitivity to pain.

Following the completion of sensory testing for each method, a feasibility score was assigned by RMC to indicate the ease of data collection. Feasibility scores ranged from 0, indicating there was no problem in collecting QST data, to 5, indicating that it was impossible to collect QST data. Table 1 describes the rubric used for assigning feasibility scores (27).

**Table 1:** Feasibility scoring rubric (27).

Feasibility score	Description
0—No problem	Minimum restraint needed; excellent cooperation; clear reaction to stimuli
1—Mild difficulty	Mild restraint needed; good cooperation; clear reaction to stimuli
2—Moderate difficulty	Moderate restraint needed; good cooperation >50% of the time; mild sensitivity of feet being touched; mild variation in reaction to stimuli
3—Significant difficulty	Significant restraint needed and resisted lateral recumbency; good cooperation <25% of the time; moderate sensitivity to feet being touched; moderate variation in reaction to stimuli
4—Extreme difficulty	Constant restraint required; not cooperative; unclear reaction to stimuli; not confident in data collected
5—Impossible	Could not collect data due to the dog's disposition and/or lack of confidence in the reactions seen due to the stimulus

QST, quantitative sensory testing.

The feasibility scoring rubric was used for evaluation of the ease with which mechanical and thermal QST data was able to be collected from dogs.

#### 2.4.1. Electronic von Frey

**Figure 2:**
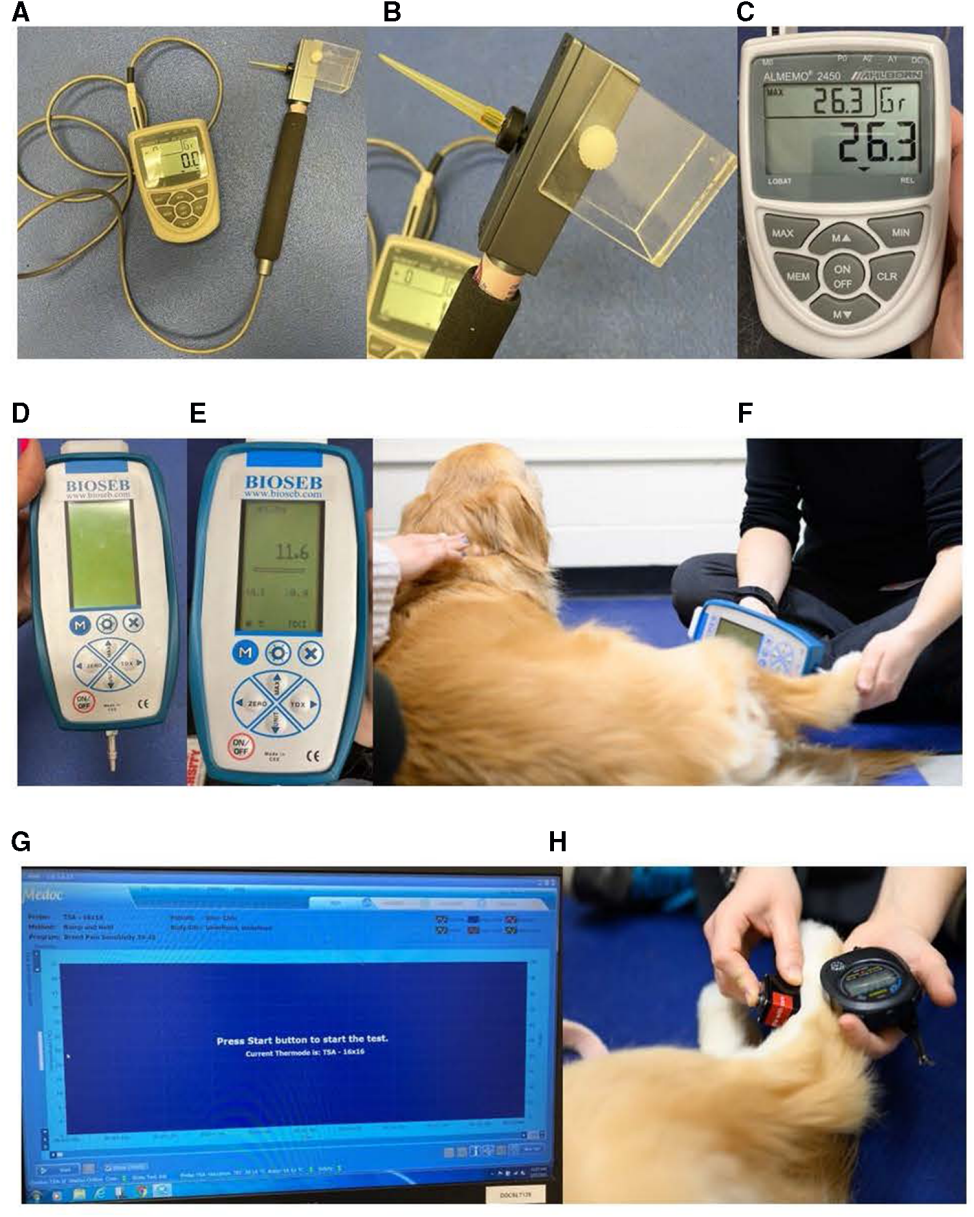
Quantitative sensory testing methods used (27). (**A–C**) EVF device: (**A**) EVF device set up displaying the von Frey tip with the cord affixed to the recording device, (**B**) close-up of the 0.9-mm von Frey tip applicator used, and (**C**) close-up of the recording device that demonstrates the current force (center), the maximum force applied (upper left), and the unit force measured (upper right). (**D–F**) PA device: (**D**) PA device setup displaying the blunt probe affixed to the recording device, (**E**) close-up of the recording device displaying the maximum force applied (center) and the unit force measured (top), and (**F**) application of the blunt probed PA to the metatarsus of the dog demonstrating the researcher's technique of applying the tip perpendicular to the dog's skin. (**G**, **H**) Thermal device including the thermosensory analyzer connected to the laptop and the thermode: (**G**) laptop screen displayed when the thermosensory analyzer is ready to start a new test and (**H**) application of the thermal probe to the metatarsus of the dog. The researcher uses a stopwatch to record the latency for the dog to display a behavioral response within one-hundredth of a second. EVFG, electronic von Frey; PA, pressure algometer.

As described previously by Knazovicky et al. (12) and Cunningham et al. (27), the EVF device (IITC model Almemo 2450; IITC Life Sciences Inc., Woodland Hills, CA, United States) consisted of a 1,000-g internal load cell with a rigid 0.9-mm von Frey tip applied (Figure 2). The amount of force applied was measured and displayed with a resolution of 0.1 g. The predetermined maximum force (considered the maximum safe force) applied was 1,000 g.

#### 2.4.2. Pressure algometer

The blunt probed PA (SMALGO algometer; Bioseb, Vitrolles, France) was securely fitted with a flat 3-mm diameter tip (12) (Figure 2). The probe was applied perpendicular to the site being tested with steadily increasing force (∼20 g/s) (27). The amount of force applied was measured and displayed with a resolution of 0.1 g. The maximum safe force applied was 2,500 g.

#### 2.4.3. Thermal probe

The thermal device (Thermal Sensory Analyzer-II; Medoc Ltd., Advanced Medical Systems, Ramat Yishai, Israel) included a thermosensory analyzer that was connected to a laptop using a USB cable, as well as a 16-mm × 16-mm thermode (27) (Figure 2). The temperature of the thermode could be varied by the controller between 0°C and 50.5°C. The program protocol used for this study is outlined in Table 2 (27). A stopwatch was used to record the latency to withdraw within one-hundredth of a second. The thermal probe was applied for a maximum of 20 s to avoid tissue injury.

**Table 2:** Program details for the thermal probe (27).

Parameters	Input
Method	Ramp and hold
Sequence	1
Baseline	39
Time before sequence (s)	0
Trigger	Auto
Destination temperature (°C)	49
Destination rate	8
Destination criterion	Temperature
Duration time (s)	30
Return option	Baseline
Return rate	1
Number of trials	1

### 2.5. Novel object task

**Figure 3:**
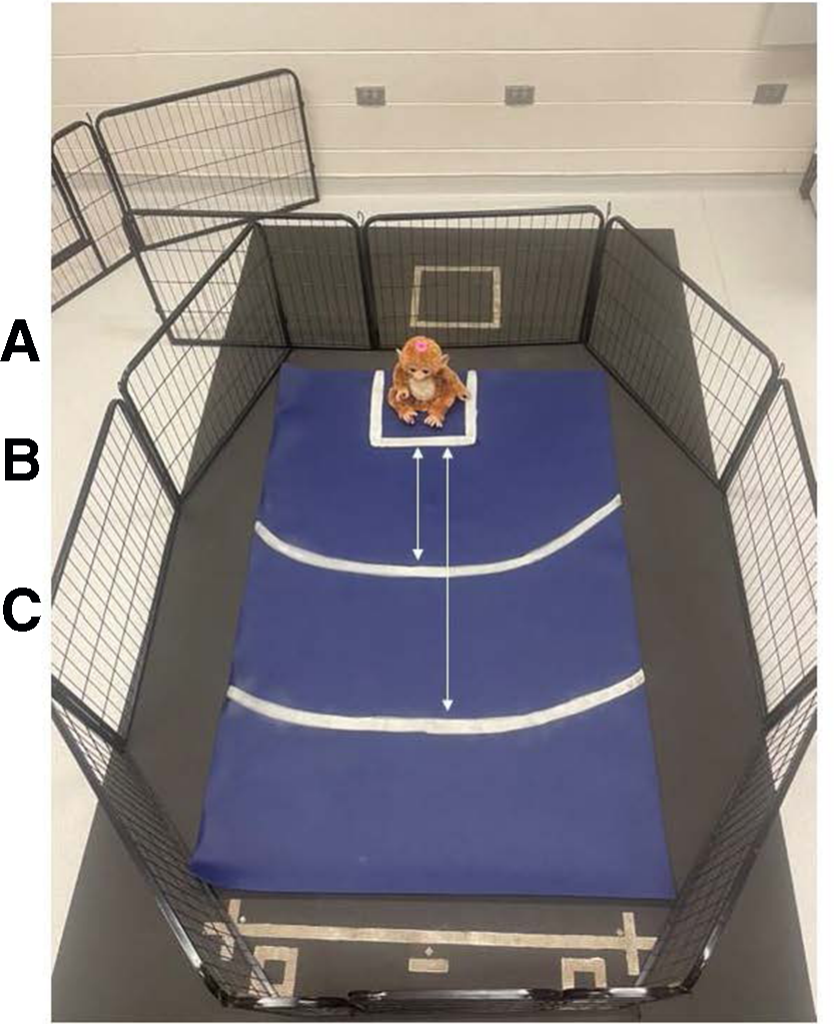
Exercise pen setup for the novel object task. Markings on the yoga mat were used to indicate where the novel object was set by the experimenter and different distances from the novel object that were used to assist observers with behavioral data coding from video recordings. (**A**) Square markings (33 cm × 33 cm) where the novel object was set by the experimenter. (**B**) Arced line indicating a distance of 50 cm from the novel object. (**C**) Arced line indicating a distance of 100 cm from the novel object.

The novel object task measured the dog's response to a stimulus that the dog was unlikely to have encountered previously, an interactive plush monkey that made noise and moved (FurReal Friends Cuddles—My Giggly Monkey; Hasbro Inc., Pawtucket, RI, United States). The handler walked the dog into a 4.75-m × 4.83-m dedicated testing room in which a dog exercise pen was set up. The dog exercise pen was positioned (∼2.16 m × 1.87 m, 61 cm in height) to surround a large yoga mat (1.83 m long, 1.22 m wide, 0.635 cm thick; Gorilla Mats Premium Large Exercise Mat; Yom Gorilla Mats, Carpinteria, CA, United States) with markings indicating where the novel object was set by the experimenter (Dr. Rachel M. P. Caddiell, henceforth referred to as RMPC) and lines that measured different distances from the novel object (Figure 3). Within the room, a white noise machine (Yogasleep Dohm Classic Sound Machine, DOHM DS; Marpac, Kings Grant, NC, United States) was used to minimize auditory distractions. Two video cameras (Panasonic Video Camera, HC-V180; Panasonic Global, Newark, NJ, United States) were positioned outside the exercise pen on tripods to ensure the dog's behavior could be visualized without blind spots from video recordings. The handler walked the dog into the exercise pen and held their collar until the experimenter turned the novel object on and set it into position. Once the novel object was set into position, the handler released the dog's collar and stepped back against the wall (outside the pen). The experimenter immediately stepped back against the wall (outside the pen) for the duration of the task. Both researcher and experimenter did not make eye contact with the dog and fixed their gaze on the floor. The experimenter was responsible for monitoring the time of the task using a stopwatch. The dog was allowed to explore the exercise pen including the novel object for 90 s. After 90 s had passed, the experimenter turned off the novel object and removed it from the exercise pen. At this time, the dog was given the option to greet the novel object, held by the experimenter, if they chose, and was able to exit the exercise pen.

Behavioral data were coded from video recordings using a continuous sampling method. The dependent measures assessed were latency to first approach the novel object, latency to vocalize, and percentage of time the dog spent oriented toward the novel object (Table 3). Additionally, a subjective scoring system was used to capture a global perspective of each dog's behavior in response to the novel object (Table 3). All continuous sampling data were collected by a single researcher (RMPC) using VLC Media Player (Version 3.0.1, VideoLan, France). An additional observer coded 20% of videos for reliability. The researcher (RMPC) trained the additional observer and inter-observer reliability was assessed prior to data collection in which both individuals scored the same eight videos (Cronbach's *α* ≥ 0.9). The inter-rater reliability was consistently excellent across the entire data collection period (Cronbach's *α* ≥ 0.9).

**Table 3:** Ethogram and subjective scoring system used to assess canine behavior for the novel object task.

Ethogram of canine behavior		
Behavior	Definition	Calculation
First approach	Record the time at which the novel object is first approached, defined as two front paws within 50 cm (white arc closest to the toy)	Time of the first approach subtracted from the start time of the encounter; expressed in seconds
First vocalization	Record the first time that the dog vocalizes. This can be a groan/growl, whine, yelp/bark, or howl	Time of the first vocalization subtracted from the start time of the encounter; expressed in seconds
Orientation toward the novel object	Total duration of time that the dog's head is facing toward the novel object	Total duration of time the dog oriented toward the novel object divided by the total time of the encounter (90 s); expressed as the proportion of time
Reaction scoring system		
Reaction score	Description	
1—Interested	Calm; expressed interest in the novel object; engaged (touches, sniffs, plays) with the novel object	
2—Apprehensive	Nervous; spent majority of time half pen away from the novel object; did approach the novel object at least once—may have jumped or flinched in response to the novel object	
3—Avoidant	Nervous; spent majority of time half pen away from the novel object; never approached the novel object; may have attempted to and/or escaped from exercise pen	

The behaviors evaluated using the ethogram included measuring the latency to perform a behavior and/or the proportion of time that an individual spent performing a behavior during the novel object task. Behavioral data were collected using continuous sampling of video recordings. The start time and end time of the encounter were recorded to calculate dependent variables for data analysis. The subjective scoring system was used to assign each dog a global reaction score for its response to the novel object. Scores were assigned after video recordings were viewed in their entirety.

### 2.6. Disgruntled stranger test

This ERT was used to measure the dog's response to a disgruntled stranger. The handler walked the dog outside of the dedicated testing room used for the novel object task and into an open hallway where an individual who the dog had not previously interacted with sat in a chair ∼2.45 m away. The stranger was wearing a hooded sweatshirt and began speaking loudly into their phone using a prepared script, facing but not looking directly at the dog. During the encounter, the dog was able to explore the area while on a leash. Immediately following the end of the script, the stranger would remove their hood and greet the dog in a friendly tone of voice. This encounter lasted for a total of 30 s. A video camera with a wide-angle lens (Panasonic Video Camera, HC-V180; Panasonic Global, Newark, NJ, United States) recorded the encounter.

Again, behavioral data were coded from video recordings. Scoring systems were used to assign each dog an initial response score and approach score (Table 4). Additionally, observers recorded whether the dog's face was oriented to the handler at any time during the encounter. This was scored as either “0”—No occurrence or “1”—Dog was oriented to the handler. All continuous sampling data were collected by a single researcher (RMPC) using VLC Media Player (Version 3.0.1, VideoLan, France). An additional observer coded 20% of the videos. The researcher (RMPC) trained the additional observer and inter-observer reliability was assessed prior to data collection in which both individuals scored the same ten videos (Cronbach's *α* ≥ 0.95). The inter-rater reliability was consistently excellent across the entire data collection period (Cronbach's *α* ≥ 0.9).

**Table 4:** Subjective scoring systems used to evaluate canine behavior for the disgruntled stranger test.

Initial response scoring system	
Initial response score	Description
1—None	No detectable reaction (attention, turning head/ears perked was considered acceptable); the dog may have sat during the encounter; the dog did not flinch or startle
2—Startled	Flinched/startled (without lowering the body but some movement was considered acceptable including a small step back from the stranger)
3—Fearful	Crouched/ducked (characterized by downward movement of body/head) without major displacement and maintained general body orientation toward the stranger
4—Avoidant	Exhibited a rapid avoidance response away from the stranger. This could be paired with a crouch and/or a change in general body orientation
Approach scoring system	
Approach score	Description
1—Immediate	Approached stranger immediately (<3 s); the dog had relaxed body language and displayed interest in and engaged with the stranger
2—Cautious	Approached cautiously (>3 s); dog maintained close proximity to the stranger and allowed stranger's touch; may have paced, whined, or licked their lips
3—Reluctant	Approached reluctantly; the dog had multiple attempts to approach the stranger, often backing away from the stranger; frequently shifted gaze from stranger to exit; tolerated the stranger's touch; may have hidden behind the handler
4—Refusal	Refused to approach the stranger; the dog would not allow a stranger to touch them; moved away from the stranger and/or toward the exit

Scores were used to assess each dog’s initial reaction to the stranger and their approach to the stranger following the encounter when the stranger greets them in a friendly tone of voice.

### 2.7. Veterinarian and general public pain sensitivity ratings

Data were retrieved from Gruen et al. (1) to obtain both veterinarians’ and general public members’ pain sensitivity ratings for the ten dog breeds/breed types that were considered for inclusion in the present study. Additional details about the sample and survey methodology can be found in Gruen et al. (1).

### 2.8. Statistical analysis

All statistical analyses were performed using R software (R Core Team). For all continuous variables, multiple regression models were chosen; cumulative link (logit) regression models were used for ordinal variables. A logistic regression model was used for binary variables.

To determine whether the veterinarians’ or general public's pain sensitivity ratings for the dog breeds of interest explained pain sensitivity thresholds measured using QST, linear regression models were used to regress pain sensitivity thresholds for each QST method and/or application site on the mean veterinarian and mean general public pain sensitivity ratings. Comparisons were made between regression models using R^2^ (i.e., the proportion of variance explained).

Dog breed and dog size (scaled dog height × weight interaction) were both previously identified by veterinarians and the general public as important dog characteristics related to dog pain sensitivity ratings (1). Models with both breed and dog height × weight interaction, as well as main effects, were singular. Therefore, the Akaike information criterion (AIC) was used to compare regression models with either dog breed or dog size as the fixed effect and determine which variable was more explanatory of pain sensitivity thresholds across all QST methods and/or application sites. A multiple regression model was used to evaluate the effect of breed on pain sensitivity thresholds for each QST method and/or application site. Follow-up analysis of variance tests for breeds was performed with pairwise comparison *t*-tests to determine differences between breeds. Benjamini–Yekutieli corrections were performed to account for multiple comparisons. Additional linear models were used to evaluate the effect of breed on pain sensitivity thresholds for each QST method accounting for the following covariates: dog characteristics (including age, sex, and body condition score) and owner-reported dog behavior (including trainability, dog-directed fear, stranger-directed fear, nonsocial fear, and touch sensitivity).

As dog behavior was a primary interest of this study, the seven dogs initially excluded from the final sample due to behavior (as pain sensitivity thresholds were unable to be obtained using QST) were included for the analyses examining feasibility for QST methods and all ERT variables. This reintroduced one border collie, one chihuahua, two German shepherds, one Maltese, and two Siberian huskies into the data set. All seven dogs were included for analyses conducted on feasibility scores for all three QST methods. Only three dogs completed the novel object test, and two dogs completed the stranger test; therefore, their data were included in these analyses, respectively.

Cumulative logit models were used to evaluate the effect of breed on feasibility score for all three QST methods. Following analysis of variance tests for overall breed effects, pairwise comparison *t*-tests were performed to determine differences between breeds. Again, Benjamini–Yekutieli corrections were used to account for multiple comparisons. Additional cumulative logit models were used to evaluate the effect of breed on feasibility score for each QST method accounting for the following covariates: dog characteristics (including age, sex, and body condition score) and owner-reported dog behavior (including trainability, dog-directed fear, stranger-directed fear, nonsocial fear, and touch sensitivity).

Linear models with breed as the fixed effect were used to determine whether the breed influenced the dog's latency to first approach the novel object, latency to vocalize during the novel object task, and the proportion of time the dog spent oriented toward the novel object. Cumulative logit models were used to evaluate the effect of breed on the dog's subjective score for the novel object task, as well as their initial response score and approach score for the disgruntled stranger test. Finally, a logistic regression model was used to determine whether the breed affected the dog's orientation to the handler during the disgruntled stranger test. The overall effect of breed was tested using analysis of variance followed by pairwise *t*-tests to determine differences between breeds for dependent measures from ERT that differed by breed, and multiple comparisons were accounted for using Benjamini–Yekutieli corrections.

To understand whether breed differences for pain sensitivity thresholds as measured across all QST methods and/or application sites were robust when accounting for breed differences found in behavioral measures, feasibility scores for QST and ERT variables that differed by breed (proportion of time the dog spent oriented toward the novel object, subjective score for the novel object task, and the initial response score and approach score for the disgruntled stranger task) were used as covariates in additional linear models with breed as a fixed effect.

Finally, we wanted to determine whether veterinarian pain sensitivity ratings were related to ERT-dependent measures that differed by breed. Therefore, an additional linear model was used to understand whether an association existed between veterinarian pain sensitivity ratings and the proportion of time the dog spent oriented toward the novel object. Cumulative logit models were used to determine whether there was a relationship between veterinarian pain sensitivity ratings and the dog's subjective score for the novel object task, their initial response score, and their approach score for the disgruntled stranger test, respectively.

*P*-values (including adjusted *p*-values) are reported as summary statistics and should be interpreted with caution to avoid making binary decisions about statistical significance.

## 3. Results

**Figure 4:**
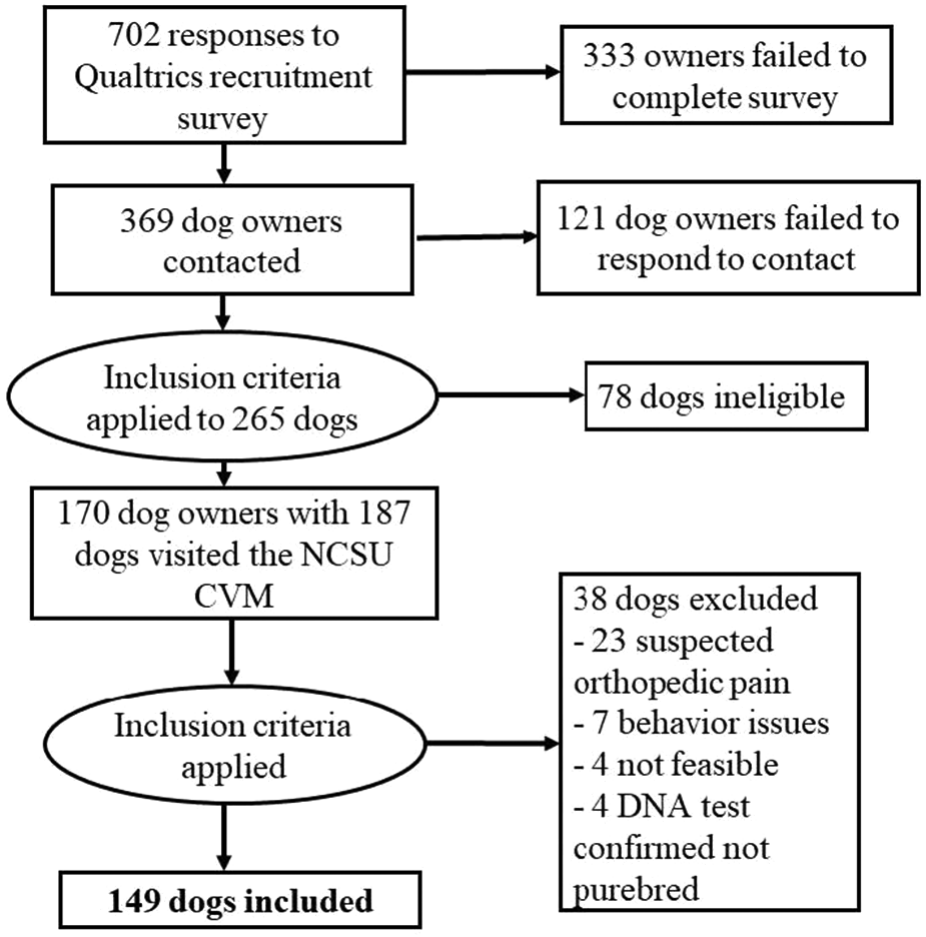
Visual representation of recruitment efforts resulting in the final sample retained.

Over the recruitment period, we received 702 responses to the screening survey. After excluding responses that were incomplete or were for dogs who did not meet inclusion criteria for breed or age, 369 dog owners were contacted for screening. Of these, 248 responded (i.e., a response rate of 67.21%). These responses were screened using inclusion criteria to ensure dogs belonged to one of the ten breeds and were of adult age for that breed, as well as that the dog(s) was considered healthy and nonpainful, which was initially determined by reviewing their veterinary records. After inclusion criterion filtering was applied, 170 dog owners with 187 dogs visited the NCSU CVM for the study. Following the screening, additional 38 dogs were excluded primarily for suspected orthopedic pain, as well as other reasons (e.g., behavior issues, not feasible to conduct QST, DNA test confirmed that they were not purebred) (Figure 4). This resulted in a final sample of 149 dogs. A summary of dog characteristics for the final sample is presented in Table 5.

**Table 5:** Characteristics of dogs represented in the final sample presented by breed/breed type.

	Breed/breed type									
	Border collie *N* = 15	Boston terrier *N* = 15	Chihuahua *N* = 14	German shepherd *N* = 16	Golden retriever *N* = 15	Jack Russell terrier *N* = 15	Labrador retriever *N* = 15	Maltese *N* = 14	Pitbull *N* = 15	Siberian husky *N* = 15
Sex										
Female spayed	4	5	6	9	7	4	4	4	9	8
Female intact	4	1	2	1	0	2	1	3	0	1
Male neutered	2	8	5	6	7	7	6	6	6	3
Male intact	5	1	1	0	1	2	4	1	0	3
Age (years)										
Range (min–max)	2–8	1–9	1–12	1–5	1–6	2–11	2–7	1–8	2–7	1–6
Mean	4.81	4.93	6.64	3.19	3.27	5.53	3.87	3.50	4.00	3.20
SD	2.03	2.87	4.07	1.33	1.49	2.53	1.64	2.41	1.46	1.32
Median	5.00	5.00	7.50	3.00	3.00	5.00	4.00	3.50	4.00	3.00
Weight (kg)										
Range (min–max)	14.2–24.5	4.8–14.8	2.1–5.7	25.4–52.5	25.4–55.5	6.1–10.9	20.2–41.5	1.8–4.8	16.6–38.6	15.1–33.7
Mean	19.17	9.71	3.31	37.22	32.33	7.65	32.89	3.07	26.61	24.08
SD	3.11	2.77	1.01	7.40	7.17	1.48	5.96	0.86	6.21	5.22
Median	18.30	10.20	3.10	36.35	30.90	7.10	35.10	2.90	24.9	24.2
Body condition score										
Range (min–max)	4–6	4–7	3–8	4–7	5–7	4–8	4–8	4–7	4–7	4–8
Median	5.00	5.00	5.50	5.00	5.00	5.00	5.00	5.00	5.00	5.00

The range, mean, SD, and median are presented for age, weight, and body condition score.

### 3.1. Correlations among QST methods

Results from the two mechanical QST methods were highly correlated (*r* = 0.715) in line with previous findings (30). However, correlations between the mechanical QST and thermal QST were low for the metatarsus (*r* = 0.358 for EVT and *r* = 0.305 for PA) and negligible for the carpus (*r* = 0.274 for EVT and *r* = 0.193 for PA); thermal thresholds were poorly correlated between the two sites (*r* = 0.360).

### 3.2. Veterinarians’ pain sensitivity ratings have minimal explanatory power for dog pain sensitivity thresholds measured by QST

Across all four QST methods and/or application sites, veterinarian pain sensitivity ratings and general public pain sensitivity ratings were negatively correlated with dog pain sensitivity thresholds. Veterinarian pain sensitivity ratings were negatively correlated with dog pain sensitivity thresholds measured by EVF [*t*(146) = −4.458, *p* = 1.64 × 10^−5^], PA [*t*(147) = −5.420, *p* = 2.38 × 10^−7^], and the thermal probe at both the metatarsus [*t*(145) = −2.092, *p *= 0.038] and carpus [*t*(143) = −1.530, *p* = 0.128]. Similarly, general public pain sensitivity ratings were negatively correlated with dog pain sensitivity thresholds measured by EVF [*t*(146) = −5.663, *p* = 7.66 × 10^−8^], PA [*t*(147) = −9.62, *p* < 2 × 10^−16^], and the thermal probe at both the metatarsus [*t*(145) = −0.909, *p* = 0.365] and carpus [*t*(143) = −1.026, *p* = 0.306]. In other words, a dog rated as being more sensitive to pain exhibited a lower pain sensitivity threshold as measured by fewer grams of force tolerated during mechanical QST or a lower latency to respond to the thermal probe.

For both mechanical devices, the general public's pain sensitivity ratings explained more of the variability than the veterinarians’ pain sensitivity ratings; the reverse was true for the thermal probe at both the metatarsus and carpus. However, across all modalities, results of the regression models found that the amount of variance explained by dog pain sensitivity ratings was relatively low (less than 20%) with the notable exception of the 38.63% of the variance in pain sensitivity thresholds measured by the PA and the general public's pain sensitivity ratings. Results for regression models are presented in Table 6.

**Table 6:** Coefficients of determination (*R*^2^) resulting from linear regression models for dog breed pain sensitivity ratings made by members of the general public and veterinarian responders with pain sensitivity thresholds measured by QST

	EVF	PA	Thermal (MT)	Thermal (C)
General public	0.180	0.386	0.006	0.007
Veterinarians	0.120	0.167	0.029	0.016

EVF, electronic von Frey; PA, pressure algometer; MT, metatarsus; C, carpus; QST, quantitative sensory testing.

### 3.3. Dog pain sensitivity thresholds differed by breed

**Figure 5:**
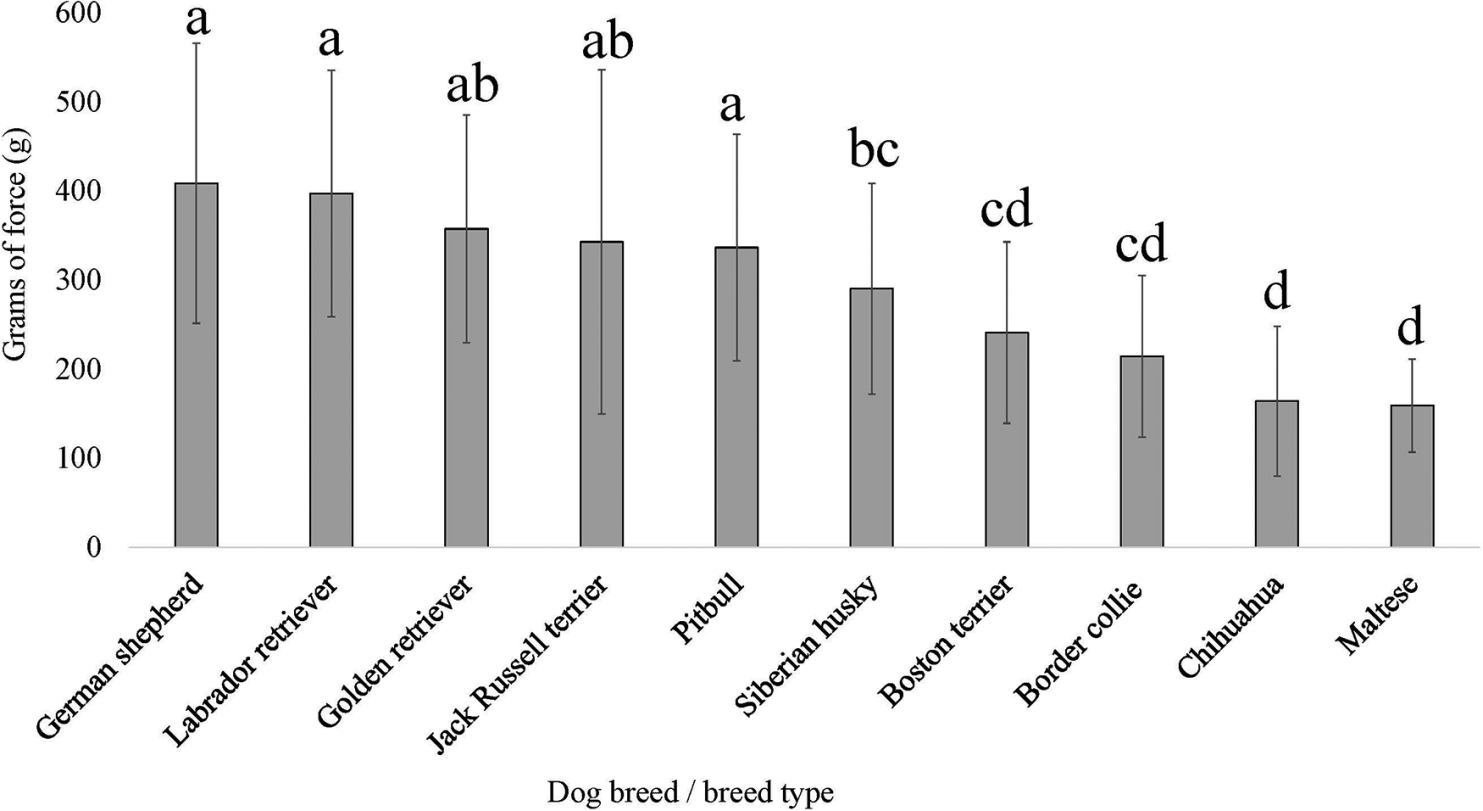
Dog breed/breed type comparisons for dog pain sensitivity thresholds for the electronic von Frey method using pairwise *t*-tests. Benjamini–Yekutieli corrections were used to account for multiple comparisons. The mean and SD values are displayed for each breed/breed type. The more grams of force the breed/breed type tolerated would indicate dogs with a higher pain sensitivity threshold or dogs who are less sensitive to pain, on average. The fewer grams of force the breed/breed type tolerated would indicate dogs with a lower pain sensitivity threshold or dogs who are more sensitive to pain, on average. Differing letters denote statistical differences between breeds at an adjusted *p* < 0.05.

**Figure 6:**
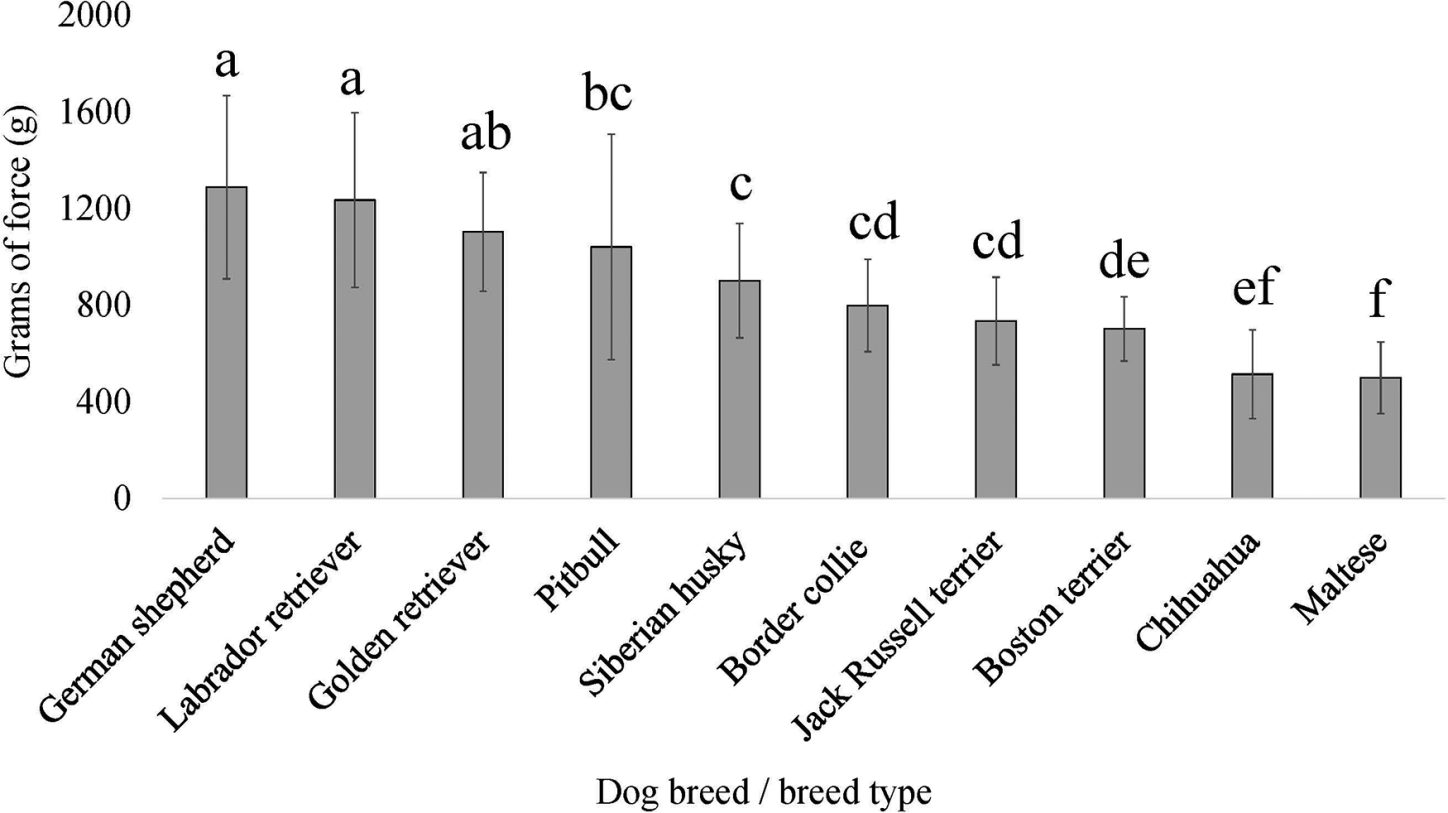
Dog breed/breed type comparisons for dog pain sensitivity thresholds for the pressure algometer method using pairwise *t*-tests. Benjamini–Yekutieli corrections were used to account for multiple comparisons. The mean and SD values are displayed for each breed/breed type. The more grams of force the breed/breed type tolerated would indicate dogs with a higher pain sensitivity threshold or dogs who are less sensitive to pain, on average. The fewer grams of force the breed/breed type tolerated would indicate dogs with a lower pain sensitivity threshold or dogs who are more sensitive to pain, on average. Different letters denote statistical differences between breeds at an adjusted *p* < 0.05.

**Figure 7:**
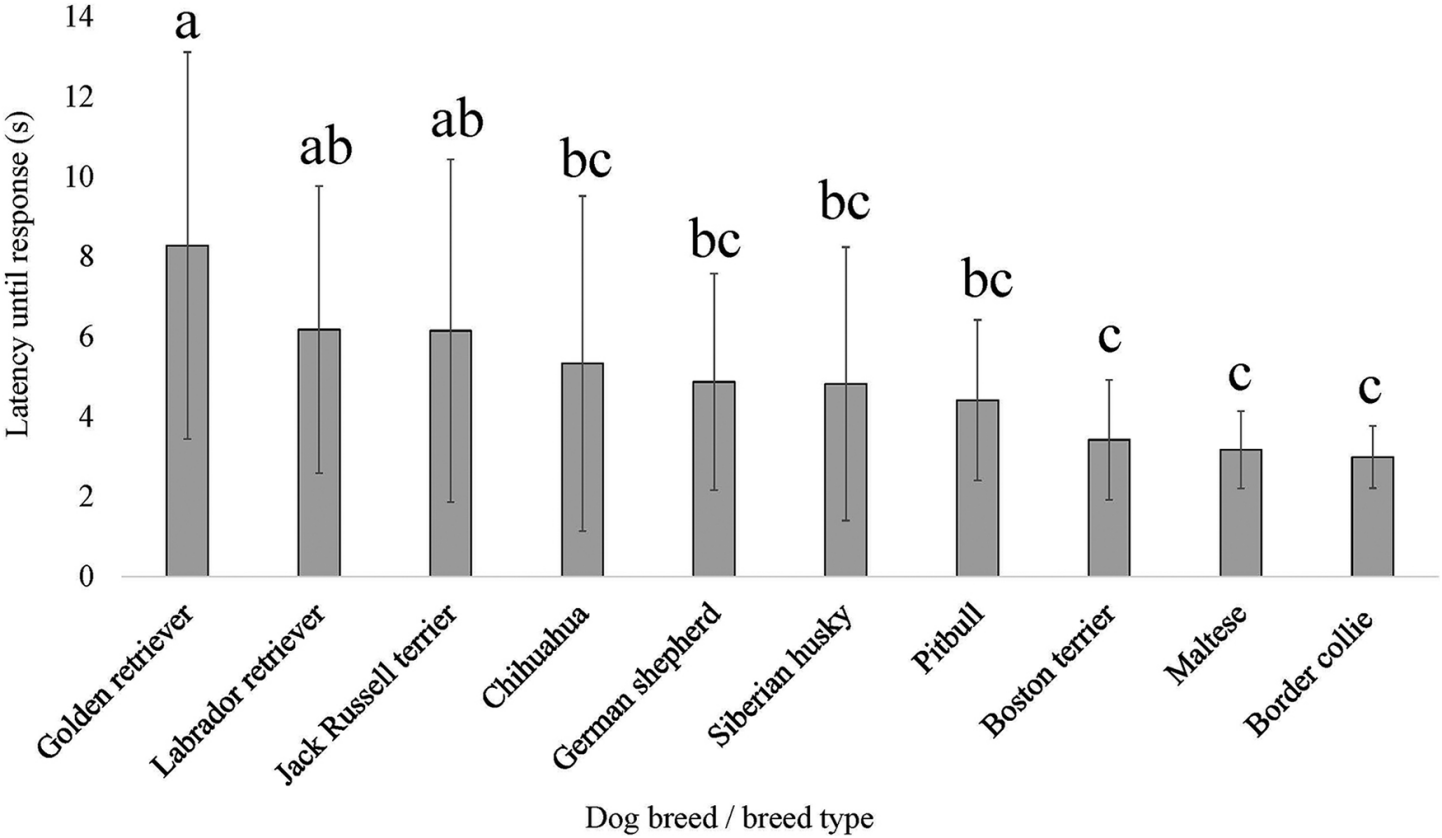
Dog breed/breed type comparisons for dog pain sensitivity thresholds for the thermal probe method positioned on the dog's metatarsus using pairwise *t*-tests. Benjamini–Yekutieli corrections were used to account for multiple comparisons. The mean and SD values are displayed for each breed/breed type. The longer latency of time the breed/breed type is allowed to pass before exhibiting a reaction would indicate dogs with a higher pain sensitivity threshold or dogs who are less sensitive to pain, on average. The shorter latency of time the breed/breed type is allowed to pass before exhibiting a reaction would indicate dogs with a lower pain sensitivity threshold or dogs who are more sensitive to pain, on average. Different letters denote statistical differences between breeds at an adjusted *p* < 0.05.

**Figure 8:**
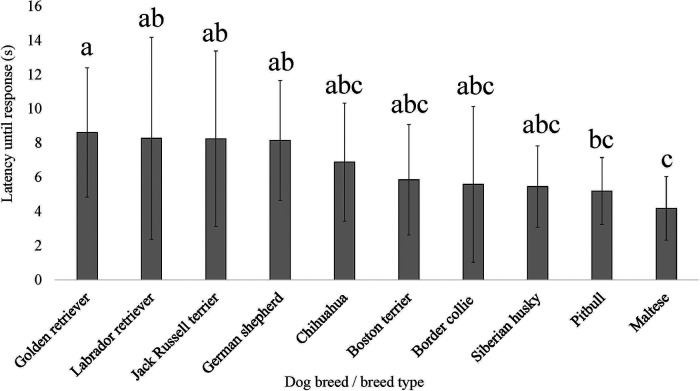
Dog breed/breed type comparisons for dog pain sensitivity thresholds for the thermal probe method positioned on the dog's carpus using pairwise *t*-tests. Benjamini–Yekutieli corrections were used to account for multiple comparisons. The mean and SD values are displayed for each breed/breed type. The longer latency of time the breed/breed type is allowed to pass before exhibiting a reaction would indicate dogs with a higher pain sensitivity threshold or dogs who are less sensitive to pain, on average. The shorter latency of time the breed/breed type is allowed to pass before exhibiting a reaction would indicate dogs with a lower pain sensitivity threshold or dogs who are more sensitive to pain, on average. Different letters denote statistical differences between breeds at an adjusted *p* < 0.05.

For three of the four QST methods and/or application sites, breed as the fixed effect provided a lower AIC compared to dog size (scaled dog height × weight interaction); therefore, the breed was generally more explanatory of dog pain sensitivity thresholds. The exception was for PA, where dog size provided the lower AIC compared to breed. There was an effect of breed on pain sensitivity thresholds as measured by both mechanical devices, EVF [*F* (9, 138) = 7.864, *p* = 2.577 × 10^−9^] (Figure 5) and PA [*F* (9, 139) = 15.256, *p* < 2.2 × 10^−16^] (Figure 6). Further, there was an effect of breed on pain sensitivity thresholds as measured by the thermal probe at both the metatarsus [*F* (9, 137) = 3.857, *p* = 2.232 × 10^−4^] (Figure 7) and carpus [*F* (9, 135) = 2.522, *p* = 0.011] (Figure 8).

The effect of breed on pain sensitivity thresholds as measured by both mechanical devices remained robust when covariates (dog characteristics and owner-reported behaviors) were considered in the model, EVF [*F* (9, 95) = 7.070, *p* = 9.026 × 10^−8^] and PA [*F* (9, 96) = 9.324, *p* = 4.68 × 10^−10^]. Again, when covariates (dog characteristics and owner-reported behaviors) were considered in the model, the breed effect remained for pain sensitivity thresholds as measured by the thermal probe at both the metatarsus [*F* (9, 94) = 3.292, *p* = 0.002] and carpus [*F* (9, 93) = 2.553, *p* = 0.011].

### 3.4. Breeds differed for feasibility scores across QST methods

Breed affected feasibility scores for all QST methods, EVF [*χ*^2^ (9) = 20.646, *p* = 0.014], PA [*χ*^2^ (9) = 22.830, *p* = 0.007], and thermal probe [*χ*^2^ (9) = 17.880, *p* = 0.037]. However, for each of these QST methods, when conducting pairwise tests on breeds and correcting for multiple comparisons, there were no differences between specific breeds (all adjusted *p*’s > 0.05) for feasibility scores. The effect of breed on feasibility scores across all QST methods remained robust when covariates (dog characteristics and owner-reported behaviors) were considered in the model, EVF [*χ*^2^ (9) = 17.692, *p *= 0.039], PA [*χ*^2^ (9) = 18.107, *p* = 0.034], and thermal probe [*χ*^2^ (9) = 19.021, *p* = 0.025].

### 3.5. Certain measures of emotional reactivity differed by breed

**Figure 9:**
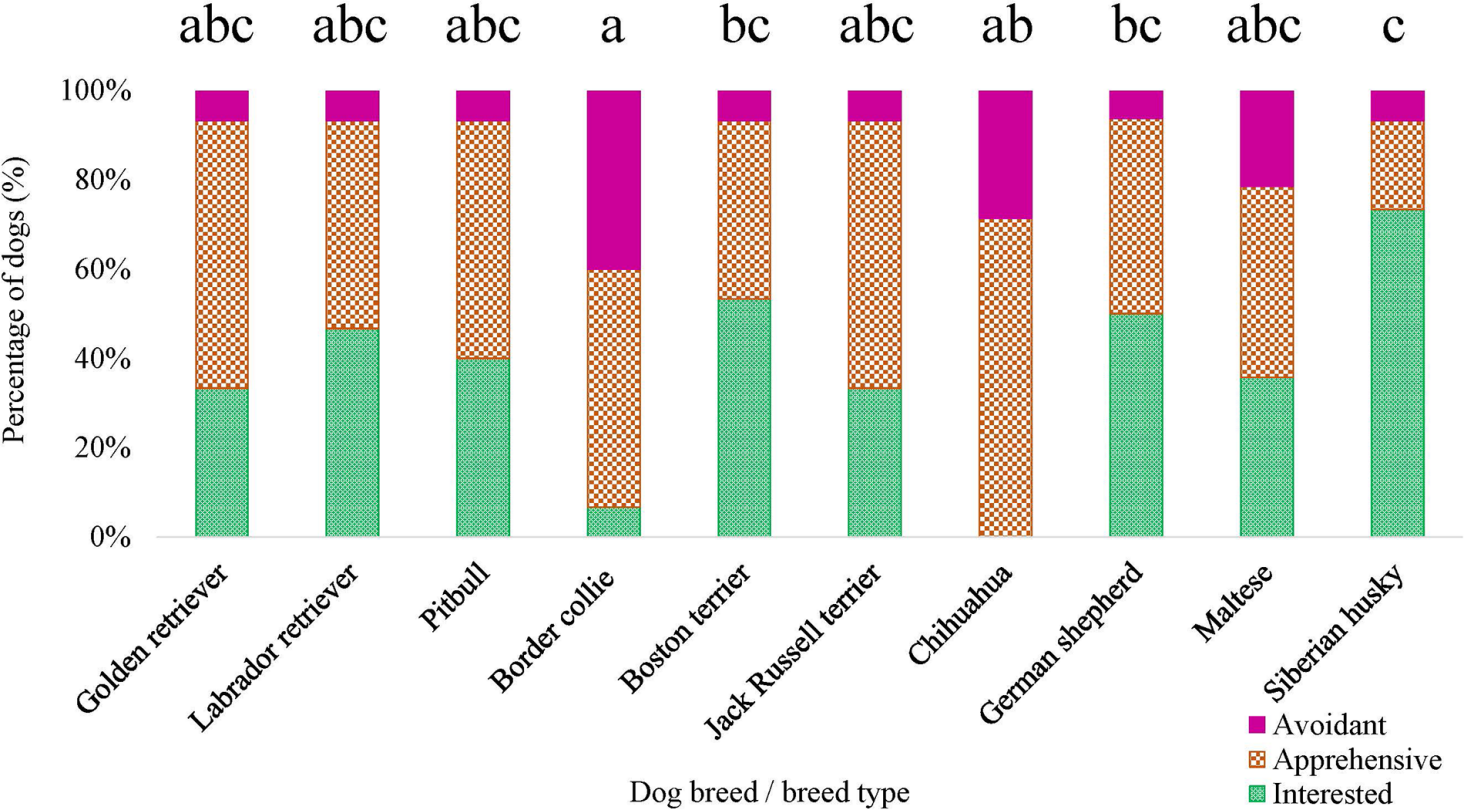
Dog breed/breed type comparisons for novel object task subjective score using pairwise *t*-tests. Benjamini–Yekutieli corrections were used to account for multiple comparisons. The proportion of dogs within each breed assigned a subjective score of 1—Interested, 2—Apprehensive, and 3—Avoidant is displayed. Different letters denote statistical differences between breeds at an adjusted *p* < 0.05.

For ERT variables measured in the novel object task, there was an effect of breed on the proportion of time the dog spent oriented toward the novel object [*F* (9, 141) = 2.441, *p* = 0.013], as well as for the dog's subjective score for the novel object task [*χ*^2^ (9) = 28.274, *p* = 8.584 × 10^−4^] (Figure 9). However, when conducting pairwise tests on breeds and correcting for multiple comparisons, there were no differences between specific breeds (all adjusted *p*’s > 0.05) for the proportion of time the dog spent oriented toward the novel object. There was no breed effect for the dog's latency to first approach the novel object (*p *> 0.05) nor the dog's latency to vocalize during the novel object task (*p* > 0.05).

**Figure 10:**
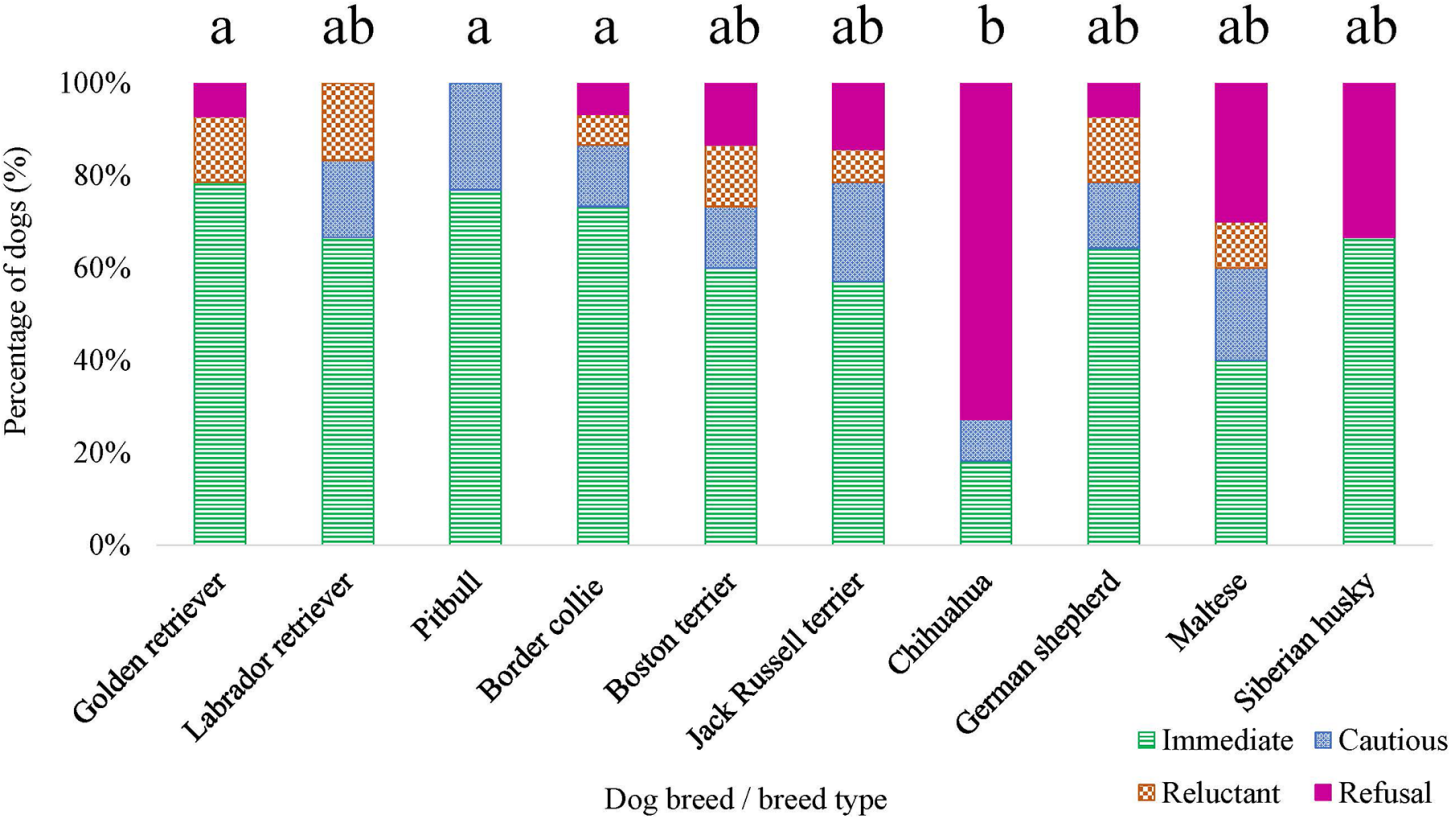
Dog breed/breed type comparisons for approach scores assigned for the disgruntled stranger test were conducted using pairwise *t*-tests. Benjamini–Yekutieli corrections were used to account for multiple comparisons. The proportion of dogs within each breed assigned an approach score of 1—Immediate, 2—Cautious, 3—Reluctant, and 4—Refusal is displayed. Different letters denote statistical differences between breeds at an adjusted *p* < 0.05.

Breed was observed to affect both the initial response score [*χ*^2^ (9) = 17.326, *p* = 0.044] and approach score [*χ*^2^ (9) = 20.261, *p* = 0.016] (Figure 10), for the disgruntled stranger test. However, when conducting pairwise tests on breeds and correcting for multiple comparisons, there were no differences between specific breeds (all adjusted *p*’s > 0.05) for the initial response score. No breed effect was detected for the dog's orientation to the handler during the disgruntled stranger test (*p* > 0.05).

### 3.6. Breed differences in pain sensitivity thresholds were robust when controlling for emotional reactivity and QST feasibility

The effect of breed on pain sensitivity thresholds as measured by both mechanical devices remained robust when ERT and feasibility variables were considered as covariates in the model, EVF [*F* (9, 103) = 8.277, *p* = 3.468 × 10^−9^] and PA [*F* (9, 104) = 13.152, *p* = 8.121 × 10^−14^]. Again, when ERT and feasibility variables were considered as covariates in the model, the breed effect remained for pain sensitivity thresholds as measured by the thermal probe at both the metatarsus [*F* (9, 101) = 3.480, *p* = 8.844 × 10^−4^] and carpus [*F* (9, 99) = 2.821, *p* = 0.005].

### 3.7. Veterinarians’ pain sensitivity ratings are positively correlated with approach scores

No relationship was detected between veterinarian pain sensitivity ratings and the proportion of time the dog spent oriented toward the novel object, the dog's subjective score for the novel object task, and the dog's initial response score for the disgruntled stranger test (*p* > 0.05). However, we found a positive relationship between the veterinarian pain sensitivity ratings and approach scores for the disgruntled stranger test, *χ*^2^ (1) = 13.243, *p* = 2.736 × 10^−4^. Veterinarians reported higher pain sensitivity ratings for dog breeds who were assigned higher approach scores (i.e., dogs who were more reluctant or avoided approaching a stranger).

## 4. Discussion

The present study found that while veterinarians’ pain sensitivity ratings were negatively correlated with pain sensitivity thresholds measured using QST (a relationship that makes intuitive sense—higher perceived pain sensitivity associated with faster responses to stimuli), these ratings provided a minimal explanation for observed differences in thresholds across breeds. Contrary to our prediction, dog breeds did differ in their pain sensitivity thresholds across QST methods, suggesting that breed differences do exist in the canine pain experience but not in the way veterinarians reported. We also found breed differences in QST feasibility and some measures of emotional reactivity (orientation to a novel object, novel object subjective score, initial response score for the disgruntled stranger test, and approach score for the disgruntled stranger test). Nevertheless, differences in pain sensitivity thresholds remained even when emotional reactivity was accounted for indicating that behavioral differences alone do not explain these findings. Intriguingly, while veterinarians’ pain sensitivity ratings did not offer much insight into observed breed differences in pain sensitivity thresholds measured in dogs, veterinarians’ pain sensitivity ratings were positively associated with dog approach scores for the disgruntled stranger test. This suggests that this particular aspect of behavior, a dog's initial greeting with a stranger, may be a factor that influences veterinarians’ ratings of pain sensitivity across dog breeds.

This is the first study to investigate breed differences in pain sensitivity thresholds across multiple modalities and methods of QST. Our finding that thermal QST differed among breeds is supported by prior research by Bowden et al. (14) that detected breed differences across three working/sport breeds (Harrier hound, Greyhound, New Zealand Huntaway) using this QST modality. Compared to Harrier hounds and Greyhounds, New Zealand Huntaways exhibited lower thermal pain sensitivity, as they took longer to exhibit a response and responded at higher temperatures. We extend the findings from Bowden et al. (14), as we included dog breeds of various sizes and historical functions, and identified additional breed differences in pain sensitivity thresholds using mechanical methods of QST. While the mechanical methods were well correlated with each other, there was a lack of correlation between the mechanical and thermal methods and within the thermal method at the two application sites. The carpus was used because this was the site used by Bowden et al. (14), and we confirmed their finding of breed differences at this site. The remaining QST was performed at the metatarsus, a commonly used site for QST with dogs (12, 13, 29, 31). Multiple methods of QST were used because these assess different nociceptive pathways and breed differences have not been fully evaluated for any QST method prior to this study. When the PA device is used to apply deep pressure at noxious ranges, the process of modulation is transmitted by C-fibers, whereas when the EVF device is used to apply punctate pressure at noxious ranges, the process of modulation is transmitted by A*δ*-fibers (11, 32). Further, when the thermal device (e.g., thermal probe set at 50°C) is used to apply heat at a consistent temperature, including when a ramping protocol is used with a steep rate of change (e.g., increase from 39°C to 50°C at a rate of change of 13°C/s), for the entire trial, the process of modulation is transmitted by A*δ*-fibers (33). These different types of pain sensitivity thresholds could be differentially related to beliefs about a breed's pain sensitivity. Despite the differences in results from the QST methods, we found that breed was the strongest predictor of differences in results for the majority of the methods, and this suggests that future work on potential biological mechanisms that may explain breed differences in pain sensitivity thresholds would be valuable. As our research group collected biological samples (e.g., blood, urine, feces) from each dog included in the current study, there is an opportunity to look for genetic differences across breeds that help determine the biological underpinnings of our findings. Additionally, we propose that future research evaluating and validating tools for pain identification and investigating pain management medication efficacy incorporate varied breeds of dogs in their study designs, analyses, and clinical recommendations.

While our results identified that breed differences do exist in pain sensitivity thresholds measured in dogs, these differences were not well explained by veterinarians’ pain sensitivity ratings for the represented breeds. This is not entirely surprising, as healthcare providers’ perceptions of patient pain often do not correspond with patient-reported pain in human medicine (34). A proposed explanation for this discrepancy is that healthcare providers’ perceptions of patient pain may be influenced by other aspects of the patient's identity (e.g., race, ethnicity, gender, socioeconomic status) that they attribute meaning to and use to inform them about patient behavior (35, 36). It is possible that veterinarians’ pain sensitivity ratings are influenced by other attributes that they associate with dog breeds, such as temperament. Many breed stereotypes described in the literature focus on perceptions of dog breeds based on temperament, personality traits, or behaviors that are associated with the breed (37–41). Ultimately, false beliefs about canine pain sensitivity have the potential to negatively impact the pain recognition and treatment of patients. Therefore, future research should focus on identifying when and how these breed-specific pain sensitivity beliefs develop in the veterinarian population.

By contrast, the general public's pain sensitivity ratings explained more of the variability in pain sensitivity thresholds as measured by mechanical QST devices. This is especially striking for the pain sensitivity thresholds measured by the PA, as the general public's pain sensitivity ratings explained 38.63% of the variance. Additionally, the PA was the only QST method in which dog size may better explain differences in dog pain sensitivity thresholds than dog breed, which likely accounts for much of the relationship between the general public's pain sensitivity ratings and the results from the PA testing. Previous studies have had mixed results when incorporating dog weight into their analyses, with some studies suggesting a correlation between mechanical pain sensitivity thresholds and weight (30, 31) and others finding no differences in mechanical pain sensitivity thresholds by weight (29, 42, 43). Moore et al. (31) found a strong positive correlation between pain sensitivity thresholds measured by EVF and dog weight and suggested scaling pain sensitivity thresholds by weight for group comparisons. However, Briley et al. (30) found low correlations between dog weight and pain sensitivity thresholds measured by PA and suggested against scaling pain sensitivity thresholds by weight; their study included dogs with a relatively restricted range of weights. To date, this is the first QST study that has looked at dog size using a scaled dog height × weight interaction. It is possible that the probe size of the PA relative to dog size may affect pain sensitivity thresholds measured by this device. Since comparisons of dog breeds cannot be balanced by weight, we would recommend future QST studies using PA to balance dog breeds across comparison groups or restrict PA use to measure pain sensitivity thresholds within the same dog over time. An alternative explanation may be that the type of afferent fiber affected during modulation by the PA device has a wider receptive range. Indeed, this may have some legitimacy because C-fibers have a wider receptive range compared to A*δ*-fibers (44). Additionally, it remains possible that nerve fiber density may contribute to the differences observed in pain sensitivity thresholds measured across QST methods. C-fiber receptive ranges are known to scale with the size of the animal (45–47), which suggests that there should not be differences in nerve density. However, as the PA device uses a larger probe size (Figure 2), it is possible that as the dog's size increases, and their C-fiber receptive range increases, the probe may not activate as many C-fibers as it would in a smaller dog. Further research is needed to investigate this theory, as C-fiber receptive ranges have not yet been documented across dogs of various sizes.

In addition to differences across pain sensitivity thresholds, we identified that breed differences exist in aspects of emotional reactivity. Previous studies have detected breed differences in canine personality traits (e.g., curiosity/fearfulness, sociability, aggressiveness, playfulness) using a battery of emotional reactivity tests (48, 49). However, the present study presents unique findings because we used dependent measures of emotional reactivity to assess specific components of fear and anxiety that dogs may experience from nonpainful aspects of being in a veterinary environment and found that breeds did differ in their behavioral responses to both the novel object task and the disgruntled stranger test. As veterinarians’ breed-specific pain sensitivity ratings offered a minimal explanation for actual pain sensitivity thresholds measured in dogs, we remained interested in whether veterinarians were influenced by other factors such as differences in behavioral reactivity. We found that breeds veterinarians reported as being more sensitive to pain were also breeds with higher (more avoidant) approach scores for the disgruntled stranger test. This suggests that the initial interaction with veterinary team members may be important in how veterinarians form their perceptions about pain sensitivity. From a clinical perspective, understanding that certain breeds may display a more reluctant and/or avoidant approach to meeting a stranger can help inform veterinary team members on how to interact with these patients, including how to best assess and treat their pain. This is particularly true because increased anxiety may influence the approach, and anxiety can increase pain intensity [as reviewed in Ref. (50)]. If anxious dogs are handled differently (with increased restraint), this may increase either their reactivity during the procedure, the intensity of their perceived pain, or both. While speculative, this would indicate that dogs with higher anxiety should be handled with an appreciation of their emotional state, low-stress handling methods, and possibly anxiolytic medications prior to handling.

To date, this is the largest study to evaluate pain sensitivity thresholds in client-owned dogs, as prior QST studies have varied in the number of subjects that they evaluated, ranging from using as few as six to as many as 30 healthy, nonpainful dogs (12–14, 29–31, 42, 43). Additionally, the present study includes the highest number of healthy, nonpainful dogs, balanced for sex, from each breed compared to prior QST studies (12–14, 29–31, 42, 43), providing sufficient power that allowed us to detect breed differences in pain sensitivity thresholds. Further, our research group made intentional efforts to hold constant environmental and personnel factors that might have influenced QST results, as evidenced by using a dedicated testing room, using female researchers and handlers, and having the same researcher (RMC) apply QST methods across modalities and application sites for all dogs.

Nevertheless, this study is not without limitations. Quantitative sensory testing remains a proxy measure of pain sensitivity thresholds for dogs, as they cannot verbally report their pain. In humans, QST endpoints are generally assessed using verbal reports, indicating that they detected the sensation or experienced discomfort (51). However, we cannot be completely certain that the evoked behavioral reactions were due to the stimulus applied to reach a noxious level. Care was taken to ensure responses were not simply reflexive movements (e.g., twitching of the paw, withdrawal of the limb before pressure or heat is applied) but rather that there was a conscious perception of each response. For dogs who appeared to exhibit reflexive movements upon contact with QST devices on their skin, the researcher would briefly touch or very gently rub the skin of the testing site prior to the QST device making contact or applying contact with the QST device without applying pressure or heat to desensitize the skin to touch before applying pressure or heat [procedures outlined in Cunningham et al. (27)]. An additional study limitation is that all four dogs who were excluded due to feasibility belonged to the same breed (chihuahua). With these dogs, the researcher could not reliably apply the probe to the dog's skin, as the QST devices frequently slipped off. This challenge was due to the anatomy and limited surface area on the metatarsus and carpus in these smaller dogs. Prior QST studies have not reported encountering this issue; however, this may be because these studies recruited larger dogs (ranging from 4.2 to 50.2 kg) (12, 29–31, 42). The inclusion of a novel object with facial features had the potential to elicit a social response. While the approach score was significantly associated with the breed in the disgruntled stranger test, the latency to approach (the closest comparative measure) was not different among breeds in the novel object test. However, the potential for a social response affecting dogs’ behavior in the novel object test should be evaluated further, and future studies may wish to avoid this confusion by using a novel object without facial features.

During recruitment for this study, care was taken to ensure that dog breeds/breed types were balanced by sex and that dogs were considered to be adults for their breed. For each breed, there was a balanced ratio of female to male dogs. However, it became difficult to balance for sex when considering spay/neuter status vs. intact. For example, nine female spayed and six male neutered pit bulls participated in the study, which means that our sample lacked representation of intact pit bulls. Some breeds were better balanced in this regard, such as the border collie in which four female spayed, four female intact, two male neutered, and five male intact dogs participated. This is a limitation of the current study, as our findings may not generalize across all sexes for each breed. Age has not previously been demonstrated to affect QST findings in dogs; however, senior dogs are more likely to have osteoarthritis, and dogs with osteoarthritis exhibit an increased pain sensitivity threshold as measured by QST (12, 29). To ensure the dogs recruited were healthy, pain-free dogs, we contacted owners with dogs within an adult age range for their breed, reviewed their medical records, and conducted a physical and orthopedic exam. If the medical records or examination findings revealed suspected orthopedic disease and/or pain, the dog was excluded from the sample. For certain breeds (chihuahua, Jack Russell terrier), finding owners willing to participate in the study with purebred dogs presented a challenge and we extended the age range to obtain the needed number of dogs per breed. Medical records and examination findings ultimately determined whether the dog could be included in the sample. Dogs with suspected orthopedic disease and/or pain were always excluded from the sample. Furthermore, as breeds were selected for the study based on findings from Gruen et al. (1), the breeds chosen primarily represent a variety of clades (e.g., breeds believed to derive from the same ancestor) and breed groups identified by the American Kennel Club (52, 53), although not all clades and breed groups were represented. Therefore, the present findings may lack generalizability to other purebred dogs and mixed-breed dogs.

## 5. Conclusions

In summary, we found that dog breeds do differ in pain sensitivity thresholds measured across QST methods; however, these differences did not fully align with the breed-specific pain sensitivity beliefs reported by veterinarians. Understanding that differences in canine pain sensitivity do exist among dog breeds is important, as it highlights a need to investigate biological mechanisms that may explain these differences and inform the assessment of analgesic efficacy across breeds. From a clinical perspective, this information may one day help clinicians tailor more effective approaches to managing canine pain within specific breeds. However, false beliefs about canine pain sensitivity present concerns because these beliefs could impact pain recognition and treatment of patients based on breed status. Future research should focus on when and how these breed-specific pain sensitivity beliefs developed in veterinarians and further evaluate what features of dogs or dog breeds contribute to these beliefs.

## Data Availability

The raw data supporting the conclusions of this article will be made available by the authors, without undue reservation.
